# Assumed White Blood Cell Count of 8,000 Cells/μL Overestimates Malaria Parasite Density in the Brazilian Amazon

**DOI:** 10.1371/journal.pone.0094193

**Published:** 2014-04-10

**Authors:** Eduardo R. Alves-Junior, Luciano T. Gomes, Daniele Ribatski-Silva, Clebson Rodrigues J. Mendes, Fabio A. Leal-Santos, Luciano R. Simões, Marcia Beatriz C. Mello, Cor Jesus F. Fontes

**Affiliations:** 1 Julio Müller Hospital, Federal University of Mato Grosso, Cuiabá, Mato Grosso, Brazil; 2 Biomedicine Department, Univag University Centre, Varzea Grande, Mato Grosso, Brazil; 3 Medical Department, Facimed Course of Medicine, Cacoal, Rondônia, Brazil; Université Pierre et Marie Curie, France

## Abstract

Quantification of parasite density is an important component in the diagnosis of malaria infection. The accuracy of this estimation varies according to the method used. The aim of this study was to assess the agreement between the parasite density values obtained with the assumed value of 8,000 cells/μL and the automated WBC count. Moreover, the same comparative analysis was carried out for other assumed values of WBCs. The study was carried out in Brazil with 403 malaria patients who were infected in different endemic areas of the Brazilian Amazon. The use of a fixed WBC count of 8,000 cells/μL to quantify parasite density in malaria patients led to overestimated parasitemia and resulted in low reliability when compared to the automated WBC count. Assumed values ranging between 5,000 and 6,000 cells/μL, and 5,500 cells/μL in particular, showed higher reliability and more similar values of parasite density when compared between the 2 methods. The findings show that assumed WBC count of 5,500 cells/μL could lead to a more accurate estimation of parasite density for malaria patients in this endemic region.

## Introduction

Microscopic examination of blood collected from patients infected with *Plasmodium* is still the most commonly used method to diagnose and estimate parasite density in malaria infections, owing to its low cost and simplicity of implementation. Quantification of parasite density is an important component in the diagnosis of malaria infection, as it helps to define the severity of the disease, assess the therapeutic response *in vivo*, and explore the effectiveness of new antimalarial drugs [1]. The accuracy of this estimation varies according to the method used; it is higher with quantification by real-time polymerase chain reaction (PCR) analysis of circulating *Plasmodium* DNA in a known volume of blood and lower for parasite quantification by microscopy of blood smears [2]. In the latter method, parasite density is inferred from the number of white blood cells (WBC) per μL of blood, which is automatically calculated using blood cell counters or assumed at a fixed value of 8,000 cells/μL, according to the World Health Organization (WHO) guidelines [1].

It is already know that a more accurate estimate of the malaria parasite density considers the variations in WBC count occurring in different age groups and also in malaria-endemic regions [3]. In order to achieve this, the use of an automated WBC count in infected patients is necessary. This allows a more accurate estimation of parasite density when compared to the method that assumes a fixed value of 8,000 cells/μL. This is due to the fact that the number of leukocytes detected in a sample of patients is lower or higher than the assumed value [4]. However, the equipment necessary to perform an automated WBC count is not always available in health care services located in malaria-endemic areas, such as the Brazilian Amazon. Therefore, an assumed value of 8,000 cells/μL has been considered as an alternative for the quantification of parasitemia in patients infected with malaria in this region.

The aim of this study was to assess the agreement between the parasite density values obtained with the 2 methods (the assumed value of 8,000 cells/μL and the automated WBC count), using WBC count as a reference. Moreover, the same comparative analysis was carried out for other assumed values of WBCs, in order to establish the number of WBCs that more closely agrees with the value of parasite density obtained using the automated WBC count.

## Patients and Methods

This study was approved by the Research Ethics Committee of the Julio Muller University Hospital, Federal University of Mato Grosso (# 130,938). Patients who agreed to participate in the study gave written informed consent.

The study was carried out at the Outpatient Malaria Clinic of the Julio Müller Hospital, Federal University of Mato Grosso, in the city of Cuiabá (MT), Brazil with 403 patients who were infected with *Plasmodium vivax* and *P. falciparum*. These individuals came from endemic areas of the Brazilian Amazon and were treated in the Outpatient Clinic between 2001 and 2013.

The diagnosis of malaria infection was carried out by microscopic assessment of thick blood smears. For each patient, blood smears of approximately 1.5 cm^2^ were prepared on 2 glass slides, which were subsequently stained with Giemsa. The overall WBC count was determined for all of the samples using automated blood cell counting equipment (Sysmex XE-2100D, Kobe, Japan).

Two different microscopy experts examined the blood smears using identical microscope model, lens, and objectives (1,000× magnification). A third expert was required only when a significant discrepancy (>40%) was observed between the values obtained by the 2 microscopy specialists.

Parasite densities were expressed as the ratio between parasites and WBC in the blood smears. The parasites were counted for every 500 WBCs in each thick blood smear. The final number of parasites per μL of blood was calculated as the formula: [(*counted parasites/500WBC)* x *counted or assumed WBC/μL*]. This calculation was repeated with WBC counts between 4,000 and 7,000 cells/μL in order to identify an assumed number of WBCs leading to a parasite density that more closely agrees with that obtained using the automated WBC count.

Statistical analysis was carried out using SPSS for Windows, version 21 (IBM Corp., Armonk, NY, USA). To assess the proportion of patients with underestimated or overestimated values obtained with the different methods of estimation, the Wilcoxon signed-rank test for paired data was applied; the difference between the estimations was considered the null hypothesis. Due to heteroscedasticity the Man-Whitney and Kruskal-Wallis nonparametric tests were used to compare WBC count among patient groups.

The intraclass correlation coefficient (ICC) for absolute similarity and 95% confidence intervals (CI) was used to express the reliability between the results of parasite density estimations that were obtained using the automated count and the assumed WBC values. The ICCs (95% CI) for the different parasite density estimations were plotted on a chart, and the coefficient at the midpoint was identified. Intraclass correlation is considered the most appropriate statistical measure to quantify the similarity between quantitative measures. ICCs range between 0 and 1, with 1 indicating maximum reliability between the measures. Values >0.70 are generally accepted as showing good reliability [5].

After logarithmic transformation of the data, Bland–Altman scatter plots were created to show the relationship between the differences in the means of the parasite densities calculated with the automated and assumed WBC count methods. In this figure, the similarity between the 2 measures can be observed, which is expressed by error dispersion (i.e., the proximity of the points to 0) [6].

## Results

The 403 patients in this study were mostly adults (90%), men (80%), and infected with *P. vivax* (79.9%). They all complained of malaria symptoms such as fever, chills, headache and/or myalgia. The mean number (standard deviation) of WBCs detected in these patients was 5,605 (1,984)/μL. Characteristics potentially associated with WBC count in these malaria patients were shown in Table 1. The WBC count was similar between age and fever groups. However, the mean number of WBC was higher in non-white patients (p = 0,024).

**Table 1:** Characteristics potentially associated with WBC count among malaria patients.

Characteristics		n	WBC/μL Mean (SD)	*p*
Age group (years)	<15	27	5,863 (1,831)	0.459*
	≥ 15	373	5,918 (2,261)	
Ethnic group	White	144	6,213 (7,314)	0.023*
	Non-White	255	6,662 (7,072)	
Fever within 24 hours before	Yes	381	6,546 (7,308)	0.595*
	No	19	5,918 (2,261)	
Parasite species	*P. vivax*	322	6,636 (7,388)	0.470**
	*P. falciparum*	76	6,295 (6,585)	
	Mixed infection	5	4,735 (1,594)	

^*^ Man-Whitney test.

^**^ Kruskal-Wallis test.

The mean (95% CI) parasite density, estimated using the automated WBC count, was 7,518 (6,585–8,451) parasites/μL; for the assumed WBC values of 8,000, 7,000, 6,000, 5,500, 5,000, and 4,000 cells/μL, the mean (95% CI) parasite density was 11,142 (9,784–12,500) parasites/μL, 9,749 (8,561–10,938) parasites/μL, 8,356 (7,338–9,375) parasites/μL, 7,660 (6,726–8,594) parasites/μL, 6,964 (6,115–7,812) parasites/μL, and 5,571 (4,892–6,250) parasites/μL, respectively (Table 2).

**Table 2:** Comparison of parasite densities using automated and assumed white blood cell counts.

Parameter	Automated WBC count (cells/μL)	Assumed WBC count (cells/μL)					
		8,000	7,000	6,000	5,500	5,000	4,000
Minimum	31	40	35	30	27	25	20
Maximum	64,930	115,947	101,453	86,960	79,713	72,467	57,973
25^th^ percentile	1,442	2,568	2,247	1,926	1,765	1,605	1,284
Median	4,503	6,016	5,264	4,512	4,136	3,760	3,008
75^th^ percentile	9,446	14,568	12,747	10,926	10,015	9,105	7,284
Mean	7,519	11,143	9,750	8,357	7,661	6,964	5,571
Standard deviation	9,527	13,869	12,135	10,402	9,535	8,668	6,934
Standard error	475	691	604	518	475	432	345
Lower 95% CI of the mean	6,586	9,785	8,561	7,338	6,727	6,115	4,892
Upper 95% CI of the mean	8,452	12,501	10,938	9,375	8,594	7,813	6,250
Geometric mean	3,404	5,151	4,507	3,863	3,542	3,220	2,576

Abbreviations: WBC, white blood cell; CI, confidence interval.

**Figure 1:**
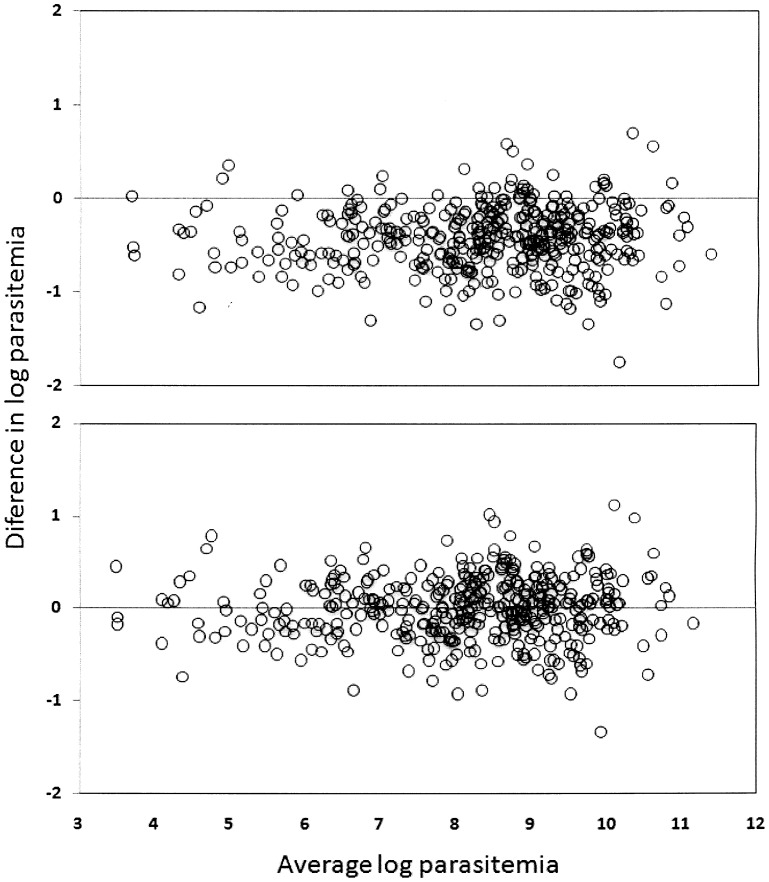
Bland–Altman plot showing parasitemia estimated by actual and assumed white blood cell (WBC) counts after logarithmic transformation. Parasite densities estimated using an assumed WBC count of 8,000/μL (A) and 5,500 cells/μL (B).

Comparisons of the results obtained using the different methods, with the automated WBC count as reference, revealed overestimated parasitemia with assumed WBC counts of 6,000, 7,000, and 8,000 cells/μL and underestimated parasitemia with counts of 5,000 and 4,000 cells/μL (Wilcoxon signed-rank test). However, no statistical difference was observed when the assumed WBC count was cells/μL (Table 3). This finding was similar to that of the Bland–Altman plot analysis, which also showed an overestimated parasite density when the assumed WBC count was 8,000 cells/μL and less variability between the 2 methods when the assumed WBC count was 5,500 cells/μL (Figure 1).

**Table 3:** Proportions of overestimated and underestimated parasite densities obtained with different assumed values of white blood cell (WBC) counts, with the automated WBC count as the reference.

Assumed WBC count (cells/μL)	Overestimated parasite density (%)	Underestimated parasite density (%)	*p* *
8,000	88.8	10.2	<0.001
7,000	78.9	19.6	<0.001
6,000	65.0	33.7	<0.001
5,500	46.4	52.1	0.33
5,000	41.2	57.3	<0.001
4,000	18.6	80.1	<0.001

* Wilcoxon signed-rank test.

Abbreviations: WBC, white blood cell.

**Figure 2:**
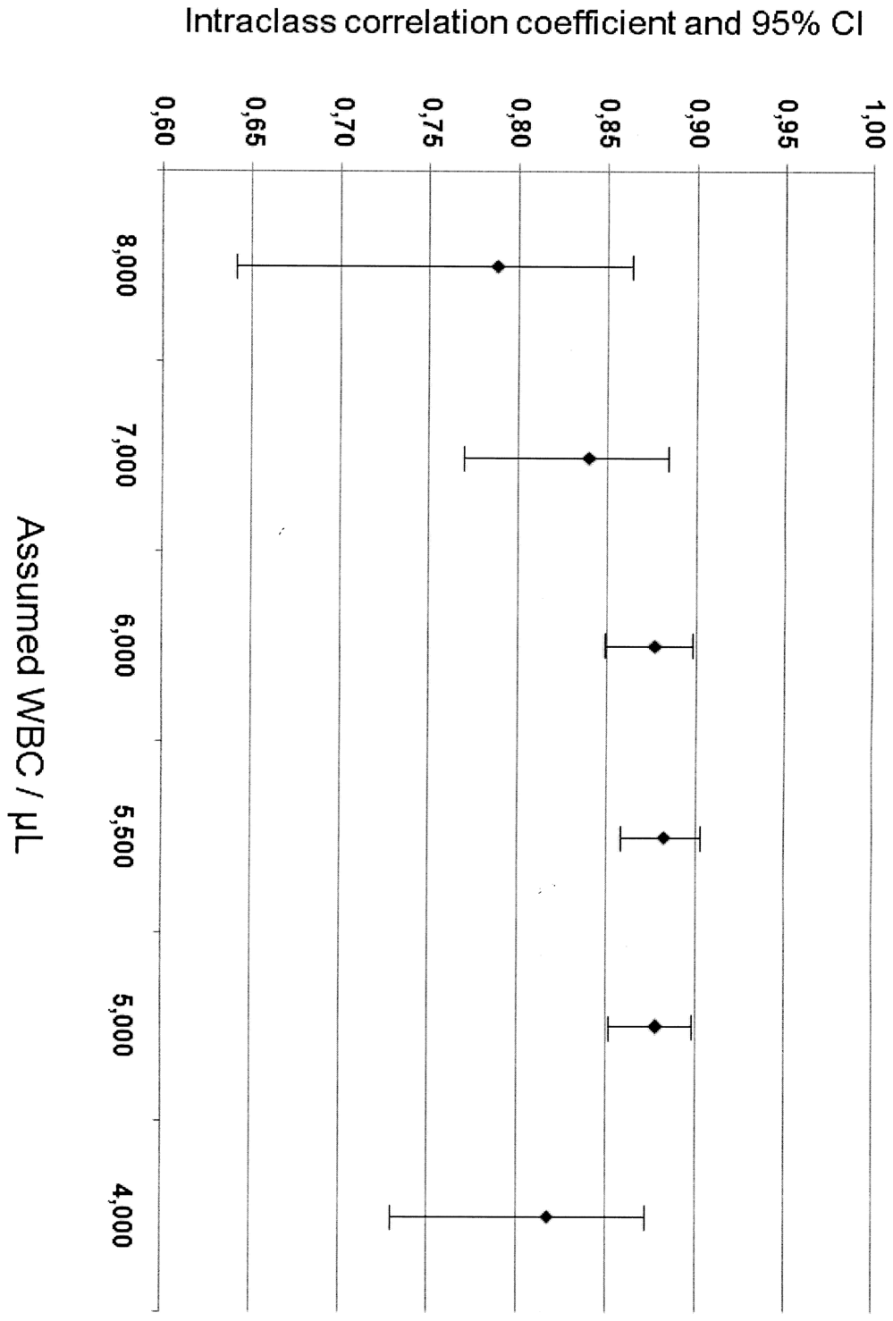
Intraclass correlation coefficients and 95% confidence intervals (CI) for different values of assumed white blood cell (WBC) counts used to estimate malaria parasite density.

The reliability analysis of the estimates of parasite density using the different assumed WBC values, with the automated WBC count as reference, revealed an increased reliability when the assumed WBC count was between 5,000 and 6,000 cells/μL. The absolute concordance was higher with assumed WBC counts of 5,000, 5,500, and 6,000 cells/μL, resulting in high ICCs (Figure 2). This analysis was stratified by ethnic group and showed the highest absolute concordance with the assumed WBC count of 5,500/μL for both white (ICC = 0.860) and non-white (ICC = 0.902) patients (data not show).

## Discussion

In this study, the use of a fixed WBC count of 8,000 cells/μL to quantify parasite density in malaria patients from endemic regions of the Brazilian Amazon led to overestimated parasitemia and resulted in low reliability when compared to the automated WBC count. Assumed values ranging between 5,000 and 6,000 cells/μL, and 5,500 cells/μL in particular, showed higher reliability and more similar values of parasite density when compared between the 2 methods. Since this cut-point is close to the mean actual WBC count, certainly it fits better the parasite density in these patients.

The fixed value of 8,000 cells/μL to estimate parasitemia in malaria was arbitrarily chosen as the reference for the average number of leukocytes in a Nigerian population in the 1950s [7]. However, it is well known that leukopenia may occur in the acute phase of malaria infection [4], [8]. Moreover, reference values for leukocyte counts vary across different ethnic groups and geographic regions of the world [9]. Therefore, determination of parasite density in malaria based on an assumed value of 8,000 cells/μL may be inaccurate for different populations.

Even in Nigeria, which was the reference country for the assumed value of 8,000 cells/μL [7], a previous study reported that parasite density was overestimated using this method in children infected with *P. falciparum* [10]. On the other hand, another study conducted with 3,044 African children with acute malaria showed that the discrepancy in the estimated parasite density using an assumed WBC count of 8,000 cells/μL was higher in younger children. In older children, a greater similarity with the automated WBC estimate was observed [11]. Therefore, the high proportion of adults in the current study explains the overestimated parasitemia using an assumed value of 8,000 cells/μL. Controversial results have also recently been published in other African countries, in which the parasite density was underestimated in children <5 years old in Ghana [12] and overestimated in pregnant women in Sudan [13], when using an assumed count of 8,000 cells/μL.

In the current study, the assumed WBC count with a parasite density that was most similar to that estimated using an automated WBC count was 5,500 cells/μL. This value is consistent with the mean (standard deviation) WBC count of 5,605 (1,984) cells/μL and the value of 6,700 cells/μL at the 75th percentile in the current sample. Considering the normal reference WBC count in the Brazilian and South American population [9], [14] and that leukopenia is typical of malaria itself [15], [16] the results of this study demonstrate that an assumed WBC count of 5,500 cells/μL better estimates the levels of parasitemia in patients infected with malaria in the Brazilian Amazon.

A potential limitation of this study is the small number of patients that prevents extrapolation of the results to other populations. Despite this, owing to the importance of an accurate determination of parasite density in malaria infection, the findings of this study demonstrate that the use of an assumed WBC count of 8,000 cells/μL [1] is not suitable for monitoring infected malaria patients in the Brazilian Amazon. In areas where an automated WBC count cannot be obtained, an assumed WBC count of 5,500 cells/μL could lead to a more accurate estimation of parasite density for malaria patients in this region. Further studies with large number of patients will be required to validate our findings.
